# Safety and efficacy of azilsartan in paediatric patients with hypertension: a phase 3, single-arm, open-label, prospective study

**DOI:** 10.1007/s10157-021-02159-9

**Published:** 2021-11-27

**Authors:** Shuichi Ito, Yuya Nishiyama, Kenkichi Sugiura, Kazuaki Enya

**Affiliations:** 1 Department of Pediatrics, Yokohama City University, Yokohama, Kanagawa Japan; 2 Takeda Development Center Japan, Takeda Pharmaceutical Company Limited, Osaka, Japan

**Keywords:** Paediatric hypertension, Azilsartan, Antihypertensive agent, Angiotensin II type 1 receptor blockers

## Abstract

**Background:**

Azilsartan is an angiotensin II receptor blocker indicated for the treatment of adult hypertension. A previous single-dose study suggested that azilsartan may also be a promising agent for paediatric hypertension. However, the long-term safety and efficacy of azilsartan in children have not been established.

**Methods:**

We conducted a phase 3, single-arm, open-label, prospective study to evaluate the safety and efficacy of azilsartan in pediatric patients with hypertension. Twenty-seven patients aged 6–15 years were treated with once-daily azilsartan for 52 weeks. The starting dose was 2.5 mg for patients weighing < 50 kg (*N* = 22) and 5 mg for patients weighing ≥ 50 kg (*N* = 5), with doses titrated up to a maximum of 20 and 40 mg, respectively.

**Results:**

Azilsartan showed acceptable tolerability at doses up to 20 mg in patients weighing < 50 kg and 40 mg in those weighing ≥ 50 kg. Most drug-related adverse events (AEs) were mild, with one patient (3.7%) experiencing a severe and serious drug-related AE (acute kidney injury). One patient (3.7%) had a mild increase in serum creatinine level, which resolved after treatment discontinuation. The blood pressure-lowering effect of azilsartan was observed as early as Week 2. Overall, approximately half of the patients achieved their target blood pressure at the end of azilsartan treatment.

**Conclusions:**

Our study suggests that azilsartan has an acceptable safety profile in hypertensive patients aged 6–15 years. Azilsartan may be a promising agent for treating paediatric hypertension.

**Supplementary Information:**

The online version contains supplementary material available at 10.1007/s10157-021-02159-9.

## Introduction

Hypertension is a common chronic disease in Japanese children and adolescents, with a detection rate of 0.5–1% in elementary and junior high school students and 3% in high school students during school health check-ups [1, 2]. As with adults, paediatric hypertension is classified as essential hypertension (with no known secondary cause) and secondary hypertension. Essential hypertension in children is associated with a high risk of organ damage, including kidney disease, cerebrovascular disorders and cardiovascular disorders, and with a high risk of hypertension tracking into adulthood [3–5]. Secondary hypertension is more common than essential hypertension in infants and younger children, with renal diseases causing 60–80% of the cases [6]. Given the complications associated with chronic hypertension, it is critical to manage high blood pressure (BP) early in paediatric patients.

The Japanese Society of Hypertension Guidelines recommends that pharmacological therapy should be considered after dietary and lifestyle changes in children with essential hypertension [6]. Meanwhile, for children with secondary hypertension, who commonly have comorbid diseases such as diabetes or chronic kidney disease (CKD), pharmacological therapy is generally recommended in the first line [6]. In patients requiring pharmacological therapy, monotherapy with an approved antihypertensive agent should be started at a low dose and increased up to the maximum approved dose until BP is normalised [6]. To date, in Japan, only five antihypertensive medications are approved for use in children: angiotensin II receptor blockers (ARBs) valsartan and candesartan; angiotensin-converting-enzyme (ACE) inhibitors enalapril and lisinopril; and calcium channel blocker amlodipine. Compared with adult hypertension, treatment options for paediatric hypertension are limited [6].

Azilsartan, approved for adult hypertension at a recommended dose of 20 mg daily (40 mg maximum), is the most commonly used ARB in Japan. In adults, azilsartan provides a greater and more sustained reduction in BP compared with that of candesartan, with comparable tolerability [7]. Furthermore, azilsartan’s efficacy is superior to that of olmesartan [8]. Previous clinical trials in the United States and Europe have indicated that the BP-lowering effects of ARBs (candesartan, cilexetil and valsartan) in children are consistent with those in adults [9, 10]. A single-dose, phase 3 study evaluating the pharmacokinetics and safety of azilsartan in Japanese children with hypertension reported no unexpected safety issues [11]. The Japanese study also showed that for the same dose, exposure to azilsartan in paediatric patients weighing ≥ 50 kg was comparable to that in healthy adults; however, in children weighing < 50 kg, the exposure was approximately twice as high [11]. Herein, we evaluate the long-term safety and efficacy of azilsartan in young patients with hypertension in Japan. On the basis of the previous pharmacokinetics study, we set the starting dose and maximum dose of azilsartan in patients weighing < 50 kg to be half of the corresponding doses in patients weighing ≥ 50 kg.

## Materials and methods

### Study design

**Fig. 1:**
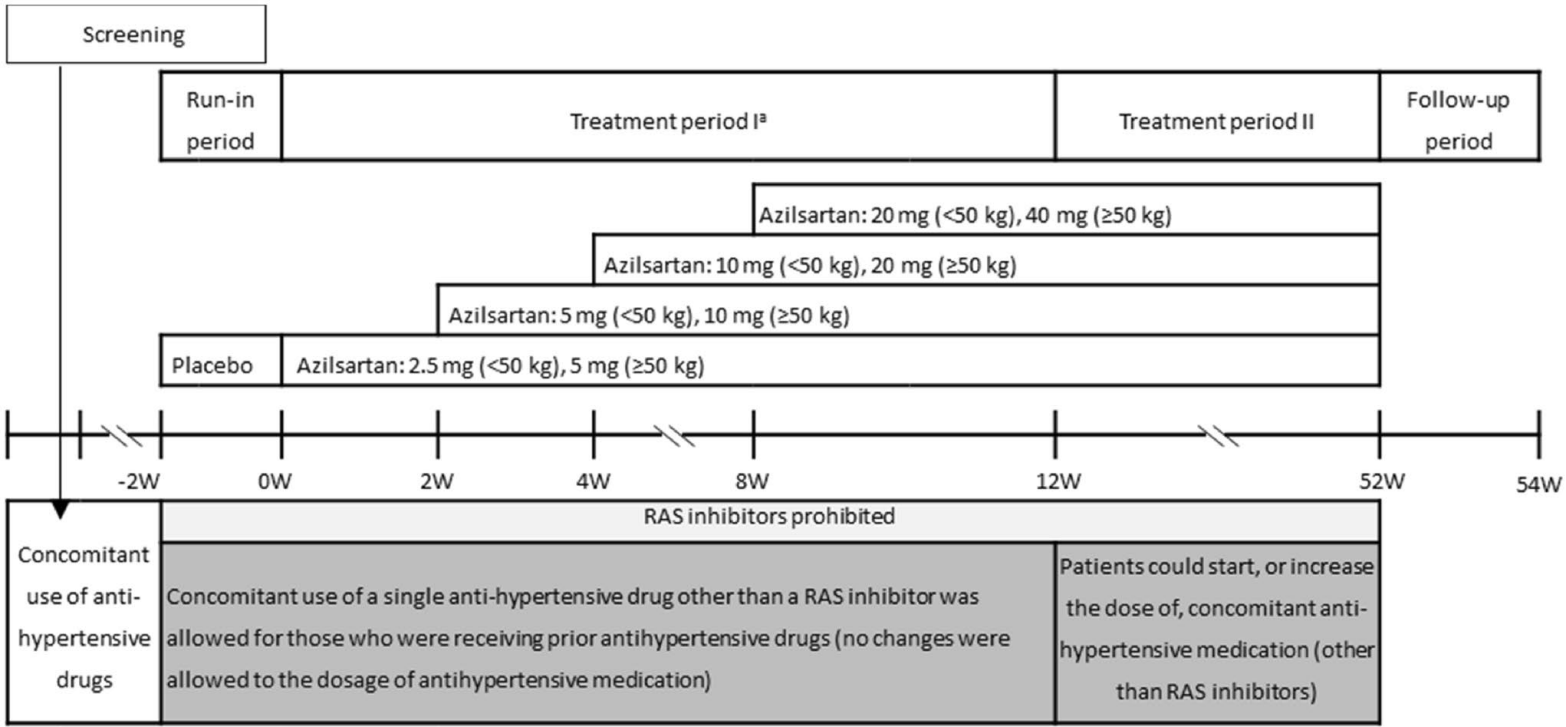
Study design. ^a^Between the visits at Week 4 and 8, an additional unscheduled visit at Week 6 may be requested at the investigator’s or subinvestigator’s discretion to titrate the dose of the study drug for further decrease in blood pressure. *RAS* renin-angiotensin-system

This study is a phase 3, open-label, single-arm, prospective study evaluating the safety and efficacy of azilsartan in paediatric patients with hypertension. The observation period was 52 weeks (Fig. 1). The study was conducted at 34 sites in Japan between August 2016 and June 2019.

### Patient population

Key eligibility criteria were: (1) aged ≥ 6 to < 16 years; (2) body weight ≥ 20 kg; and (3) a diagnosis of hypertension, defined as having office sitting diastolic or systolic BP (DBP or SBP) ≥ 95 percentile for essential hypertension without concomitant hypertensive organ damage, or ≥ 90 percentile for secondary hypertension with concomitant CKD, diabetes, heart failure or any hypertensive organ damage.

Key exclusion criteria were: (1) poorly controlled hypertension indicated by an office sitting SBP higher by ≥ 15 mmHg and/or an office sitting DBP higher by ≥ 10 mmHg than the 99 percentiles of the reference BP values of the patients by gender and age (excluded as a safety measure since concomitant use of renin-angiotensin-system [RAS] inhibitors and other antihypertensive medications is restricted in this study; see Treatment section below); (2) a diagnosis of malignant or accelerated hypertension; (3) noncompliance with the study drug during the run-in period (defined as patient receiving < 70% or > 130% of the study drug they should receive); (4) severely decreased glomerular filtration rate ([eGFR] < 30 mL/min/1.73 m^2^), is receiving dialysis, or has a renovascular disease affecting one or both kidneys, severe nephrotic syndrome not in remission, or a serum albumin level < 2.5 g/dL; (5) a history of, or signs/symptoms of serious cardiovascular, hepatobiliary, gastrointestinal, endocrine (e.g. hyperthyroidism, Cushing’s syndrome), haematological, immunological, urogenital or psychiatric disease, cancer, or any other disease that adversely affects patient’s health or potentially confounds the study results; (6) has haemodynamically significant left ventricular outflow obstruction due to aortic stenosis or aortic valvular disease, or is scheduled to undergo a medical procedure affecting BP during the study (e.g. correction of arterial anomaly); (7) a history of or concurrent clinically significant abnormality of 12-lead electrocardiogram (ECG); (8) poorly controlled diabetes indicated by haemoglobin A1c > 9.0% at screening; (9) an alanine aminotransferase or aspartate aminotransferase level of ≥ 2.5 × the upper limit of normal (ULN), or a total bilirubin level of ≥ 1.5 × ULN at screening, severely impaired hepatic function, any active liver disease, or jaundice, or (10) hyperkalaemia exceeding ULN at screening.

### Treatment

The study comprised a 2-week run-in period, a 52-week treatment period and a 2-week follow-up period (Fig. 1). Patients visited the clinic during the treatment period at Weeks 0, 2, 4, 8, 12, 16, 20, 24, 32, 40 and 52, and at the end of the follow-up period at Week 54.

During the run-in period, patients received placebo in a single-blind manner. If their BP met the inclusion criteria after a minimum of one week, the patients could enter the treatment period. For patients who had been previously treated with any antihypertensive medication and whose BP did not meet the inclusion criteria by the second week, the run-in period could be extended by up to 4 weeks.

At the start of the run-in period, patients discontinued RAS inhibitors (ACE inhibitors, ARBs, and direct renin inhibitors), down-titrating to discontinuation where needed. When the patient required down-titration, a minimum of 7 days was required between complete discontinuation and the start of the treatment period. Patients taking antihypertensive medications other than RAS inhibitors prior to starting the run-in period were allowed to continue one of these antihypertensive medications in addition to the study medication during the treatment period if deemed necessary by the investigators.

The treatment period was split into treatment period I (Week 0–12) and treatment period II (Week 12–52). At Week 0, patients weighing < 50 kg were started on an initial dose of 2.5 mg azilsartan and patients weighing ≥ 50 kg on 5 mg azilsartan, once a day. If patients had not achieved target BP (see the “Endpoints” section) and had no issues with tolerability, the dose was titrated up at the scheduled visits at Week 2, 4 and 8, and at an unscheduled visit of Week 6, as requested by investigators. Azilsartan was titrated up to 5, 10 and 20 mg in patients weighing < 50 kg and to 10, 20 and 40 mg in patients weighing ≥ 50 kg. Azilsartan was titrated down if tolerability was a concern at the discretion of the investigator. For patients treated with a single antihypertensive drug other than a RAS inhibitor at the start of treatment period I, no changes were allowed to the dosage of antihypertensive medications during treatment period I. During treatment period II, azilsartan was titrated up to the highest dose (20 mg for patients weighing < 50 kg or 40 mg for patients weighing ≥ 50 kg) to achieve target BP, given acceptable tolerability. Patients who did not achieve the target BP at the highest dose of azilsartan could start, or increase the dose of, concomitant antihypertensive medications (other than RAS inhibitors) at the investigator’s discretion. If dose reduction or interruption was deemed as necessary by the investigator due to tolerability concerns (considering the severity of adverse events [AEs], especially for renal and hepatic adverse events), any concomitant antihypertensive medications were reduced or interrupted first, and azilsartan thereafter. Each patients’ adherence rate for azilsartan was calculated by the following formula: number of pills taken (number of dispensed pills − number of leftover pills)/expected number of pills to be taken (number of days) × 100.

### Endpoints

The primary objective of this study was to evaluate the safety of azilsartan. Safety data were assessed at baseline, at visits during the treatment periods, and at the follow-up at Week 54. The safety endpoints were: (1) treatment-emergent AEs (TEAEs; defined as any AE occurring after starting azilsartan treatment); (2) anthropometric (weight, height and body mass index [BMI]) measurements; (3) laboratory tests; (4) resting 12-lead ECG; and (5) vital signs (office standing BP, office sitting and standing pulse rate, and home sitting BP). TEAEs were coded using Medical Dictionary for Regulatory Activities (MedDRA, version 21.0). A drug-related AE was defined as an AE that followed a reasonable temporal sequence from the administration of the drug, or for which a causal relationship was at least a reasonable possibility, as deemed by the investigator. TEAEs of special interest were hypotension and renal impairment. Severity of AEs was categorised as follows: mild (transient and easily tolerated), moderate (causing discomfort and interruptions in daily activities) and severe (considerably interfering with daily activities). A serious AE was defined as an untoward medical condition that was life threatening or resulted in death or hospitalisation.

The secondary endpoints were mean changes from baseline in trough office sitting SBP and DBP, and the proportion of patients who achieved target BP (sitting BP of < 95th and < 90th percentiles of the reference BP values of children by gender and age for essential and secondary hypertension, respectively) at the end of treatment period I and azilsartan treatment. All office blood pressure measurements were obtained in the morning (approximately 21–27 h after the last dose of azilsartan, except for Week 16 at which BP was measured after taking the day’s dose) using a pre-specified blood pressure monitor on a designated arm (right arm unless the left arm had higher BP at screening). The patients were instructed not to eat or bathe within one hour before the measurement, and no caffeine was allowed within 30 min before the measurement.

### Data analysis

The full analysis set (FAS) and safety analysis set (SAS) consisted of all patients who received at least one dose of azilsartan during the treatment period.

Patient baseline characteristics were analysed descriptively in the whole population, in patients weighing < 50 kg and in those weighing ≥ 50 kg. The mean and standard deviation (SD) were computed for continuous measurements, including age, body weight, BMI, disease duration, office sitting SBP and DBP, serum creatine and eGFR. Frequencies and proportions were computed for gender, type of hypertension, use of anti-hypertensives (prior and at the start of treatment period I), and presence/severity of CKD.

Safety analysis used the SAS and calculated the incidence, by group, of TEAEs, serious AEs, drug-related AEs and TEAEs of special interest.

Efficacy analyses used the FAS and total patients from each visit during the treatment period, and included summary statistics, mean and SD of change from baseline for office trough sitting SBP and DBP, and proportion of patients who achieved target BP.

### Sample size

Assuming the mean change of trough sitting DBP from bassline (Week 0) to the end of treatment period I of − 6.5 mmHg and an SD of 10.5 mmHg, and the mean change of trough sitting SBP from baseline to the end of the treatment period I of − 9.5 mmHg and an SD of 15.5 mmHg, a sample size of 50 was initially planned to achieve at least 90% power by a one-sample t-test at the 0.05 significance level (2-sided). At the end of the planned registration period of two years, patient enrolment was discontinued due to the difficulty in enrolling paediatric patients who meet the eligibility criteria, upon consultation with the Japanese authority (Pharmaceuticals and Medical Devices Agency).

## Results

### Patient flow

**Fig. 2:**
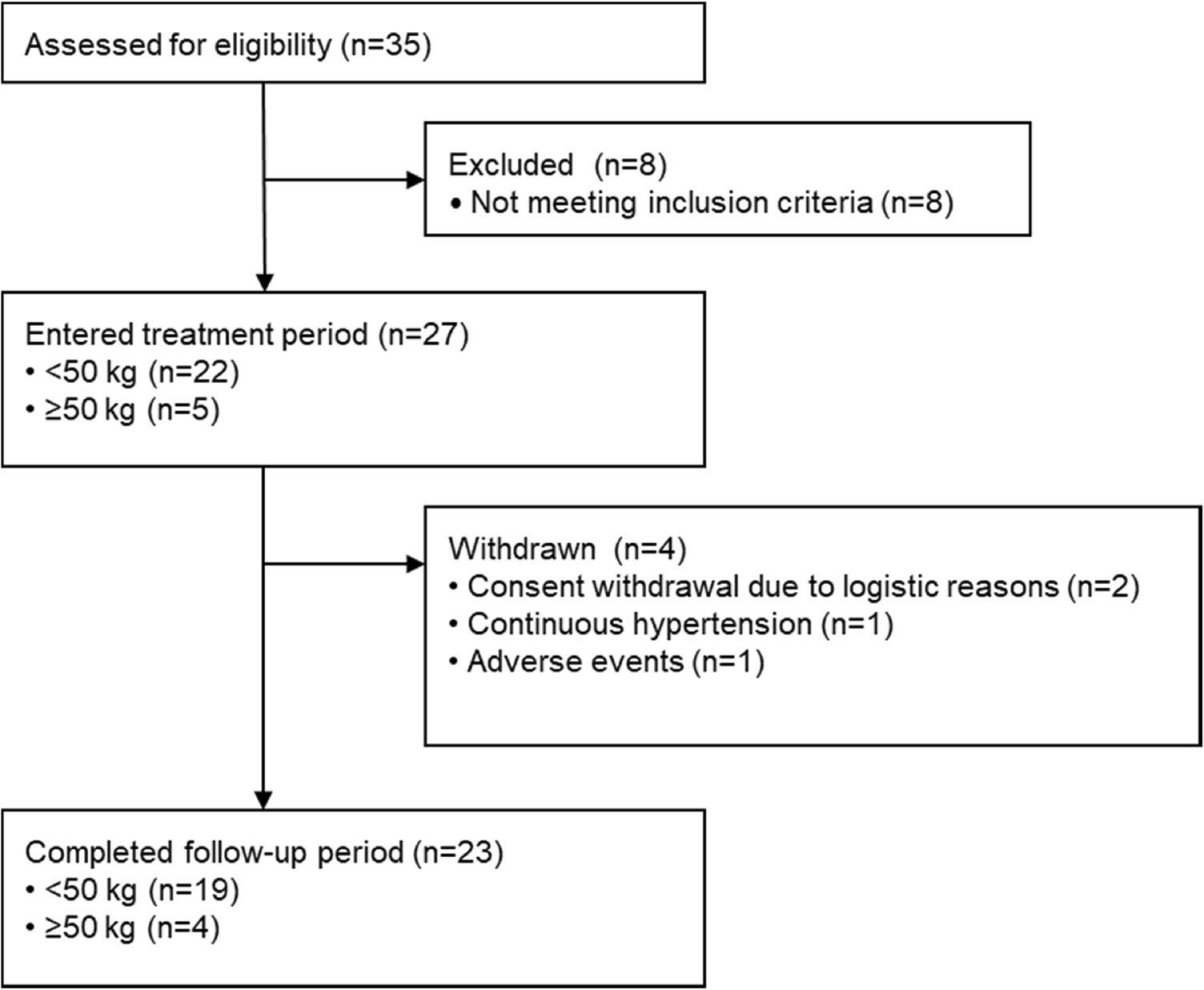
Patient flow

A total of 35 patients enrolled across 17 sites in Japan (Fig. 2). Of the 27 patients who entered into the treatment period (22 patients < 50 kg; 5 patients ≥ 50 kg), 23 patients completed follow-up (19 patients weighing < 50 kg; 4 patients weighing ≥ 50 kg). Both the FAS and SAS consisted of 27 patients.

### Patient characteristics and treatment exposure

Demographic and baseline characteristics of the patients are summarised in Table 1. The mean age (SD) of patients was 9.0 (2.5) years for the < 50 kg group and 12.8 (2.5) years for the ≥ 50 kg group. The mean duration (SD) of disease was 2.3 (2.2) years in patients in the < 50 kg group and 1.7 (2.4) years in the ≥ 50 kg group. In the total population, most (88.9%) had secondary hypertension; of those, 20 patients had renal hypertension, 1 had renovascular hypertension, and 5 had others; no patient had endocrine hypertension. Nearly three quarters (74.1%) of the total population was not receiving antihypertensive medications prior to the run-in period. At baseline, mean office sitting SBP/DBP was 123.2/72.1 mmHg in the < 50 kg group and 136.6/71.6 mmHg in the ≥ 50 kg group.

**Table 1:** Demographics and baseline characteristics of patients (SAS)

Characteristic	< 50 kg group (*N* = 22)	≥ 50 kg group (*N* = 5)	Total (*N* = 27)
Age, years	9.0 ± 2.5	12.8 ± 2.5	9.7 ± 2.9
Gender			
Male	13 (59.1)	4 (80.0)	17 (63.0)
Female	9 (40.9)	1 (20.0)	10 (37.0)
Weight, kg	31.9 ± 8.3	63.8 ± 10.1	37.8 ± 15.23
BMI, kg/m^2^	19.3 ± 4.2	24.9 ± 2.7	20.3 ± 4.5
Disease duration, years	2.3 ± 2.2	1.7 ± 2.4	2.2 ± 2.2
Type of hypertension			
Essential hypertension	1 (4.5)	2 (40.0)	3 (11.1)
Secondary hypertension^a^	21 (95.5)	3 (60.0)	24 (88.9)
Antihypertensive medications			
Prior to run-in period^b^	6 (27.3)	1 (20.0)	7 (25.9)
At the start of treatment period I	2 (9.1)	1 (20.0)	3 (11.1)
Office sitting BP, mmHg			
SBP	123.2 ± 12.6	136.6 ± 8.3	125.7 ± 12.9
DBP	72.1 ± 14.0	71.6 ± 11.9	72.0 ± 13.4
Serum creatinine, mg/dL	0.5 ± 0.2	0.6 ± 0.2	0.5 ± 0.2
eGFR, mL/min/1.73 m^2^	99.6 ± 31.0	131.4 ± 26.2	105.5 ± 32.2
CKD^c^	8 (36.4)	0	8 (29.6)
Mild	6 (27.3)	0	6 (22.2)
Moderate	2 (9.1)	0	2 (7.4)
Had kidney transplantation	4 (18.2)	0	4 (14.8)
RAS inhibitors^d^ prior to run-in period	5 (22.7)	1 (20.0)	6 (22.2)

Values are mean ± standard deviation for continuous variables and *N* (%) for categorical variables

*BMI* body mass index, *BP* blood pressure, *CKD* chronic kidney disease, *DBP* diastolic blood pressure, *eGFR* estimated glomerular filtration rate, *RAS* renin-angiotensin-system, *SAS* safety analysis set, *SBP* systolic blood pressure

^a^Underlying diseases by Medical Dictionary for Regulatory Activities System Organ Class were, < 50 kg group (*N* = 22); congenital, familial and genetic disorders (18%); injury, poisoning and procedural complications (5%); metabolism and nutrition disorders (14%); renal and urinary disorders (59%); and vascular disorder (5%); and ≥ 50 kg group (*N* = 5); metabolism and nutrition disorders (20%) and renal and urinary disorders (60%)

^b^Including renin-angiotensin-system (RAS) inhibitors in six patients (five in the < 50 kg group and one in the ≥ 50 kg group)

^c^CKD is categorized based on eGFR (mL/min/1.73 m^2^) values as follows: normal eGFR, ≥ 90; mild, 60–89; moderate, 30–59; severe, 15–29; end-stage renal disease, < 15 [12]

^d^Angiotensin-converting-enzyme, angiotensin II receptor blockers, and direct renin inhibitors

The mean duration of exposure to azilsartan was 337 and 324 days in the < 50 kg and ≥ 50 kg groups, respectively. During treatment period II, one patient (3.7%) each required addition of another antihypertensive or up-titration of a concomitant antihypertensive. Mean adherence to study medication was > 96% in each group.

### Primary endpoint: safety

TEAEs were reported in 86.4% of patients in the < 50 kg group and 100% of patients in the ≥ 50 kg group (Table 2). Most patients experienced mild TEAEs, with one patient having three severe serious TEAEs (kidney transplant rejection, complications of transplanted kidney, and acute kidney injury [AKI]; < 50 kg group), and another patient having a moderate serious TEAE (varicella; < 50 kg group). One patient discontinued treatment due to a mild TEAE (increased serum creatinine; < 50 kg group). No deaths were reported in either group. The most commonly observed TEAEs are summarised in Supplementary file 1.

**Table 2:** Overview of TEAEs (SAS)

Adverse event	< 50 kg group		≥ 50 kg group		Total	
	Events	Patients (*N* = 22)	Events	Patients (*N* = 5)	Events	Patients (*N* = 27)
Any TEAE	122	19 (86.4)	13	5 (100.0)	135	24 (88.9)
Mild	116	17 (77.3)	12	4 (80.0)	128	21 (77.8)
Moderate	3	1 (4.5)	1	1 (20.0)	4	2 (7.4)
Severe	3	1 (4.5)	0	0	3	1 (3.7)
Drug-related TEAE	11	9 (40.9)	3	3 (60.0)	14	12 (44.4)
TEAEs leading to treatment discontinuation	1	1 (4.5)	0	0	1	1 (3.7)
Serious TEAEs	4	2 (9.1)	0	0	4	2 (7.4)
Not drug-related	3	1 (4.5)	0	0	3	1 (3.7)
Drug-related	1	1 (4.5)	0	0	1	1 (3.7)
Deaths	0	0	0	0	0	0

Values are *N* (%)

*SAS* safety analysis set, *TEAE* treatment-emergent adverse event

The incidence of drug-related TEAEs was 40.9% in the < 50 kg group and 60.0% in the ≥ 50 kg group; the most commonly reported TEAEs by MedDRA preferred term were dizziness (9.1%) and headache (9.1%) in the < 50 kg group, and postural dizziness (20.0%), syncope (20.0%) and renal impairment (20.0%) in the ≥ 50 kg group (Table 3). All drug-related TEAEs were mild, except for one patient experiencing a severe, serious drug-related AE (AKI).

**Table 3:** Drug-related TEAEs (SAS)

System organ class Preferred term	< 50 kg group (*N* = 22)	≥ 50 kg group (*N* = 5)	Total (*N* = 27)
Any TEAE	9 (40.9)	3 (60.0)	12 (44.4)
Investigations	1 (4.5)	0	1 (3.7)
Serum creatinine increased	1 (4.5)	0	1 (3.7)
Metabolism and nutrition disorders	1 (4.5)	0	1 (3.7)
Hyperkalaemia	1 (4.5)	0	1 (3.7)
Nervous system disorders	4 (18.2)	2 (40.0)	6 (22.2)
Dizziness	2 (9.1)	0	2 (7.4)
Headache	2 (9.1)	0	2 (7.4)
Dizziness (postural)	0	1 (20.0)	1 (3.7)
Syncope	0	1 (20.0)	1 (3.7)
Renal and urinary disorders	2 (9.1)^a^	1 (20. 0)	3 (11.1)^a^
Renal impairment	1 (4.5)	1 (20.0)	2 (7.4)
Acute kidney injury	1 (4.5)^a^	0	1 (3.7)^a^
Vascular disorders	2 (9.1)	0	2 (7.4)
Hypotension	1 (4.5)	0	1 (3.7)
Orthostatic hypotension	1 (4.5)	0	1 (3.7)

Values are *N* (%)

*SAS* safety analysis set, *TEAE* treatment-emergent adverse event

^a^Includes one severe case. All other adverse events were mild in severity

With respect to TEAEs of special interest (related or unrelated to the study drug), TEAEs related to hypotension were reported in seven patients (five in < 50 kg group and two in ≥ 50 kg group), and TEAEs related to renal impairment were reported in two patients in the < 50 kg and one patient in the ≥ 50 kg group.

No clinically relevant changes from baseline were observed in haematology or serum chemistry parameters, except in one patient (3.7%) in the < 50 kg group who had a slight increase in serum creatinine (Table 3), which resolved after the patient discontinued treatment. There were no remarkable findings or clinical concerns with vital signs, physical examinations or ECGs during the study.

### Secondary endpoint: efficacy

**Fig. 3:**
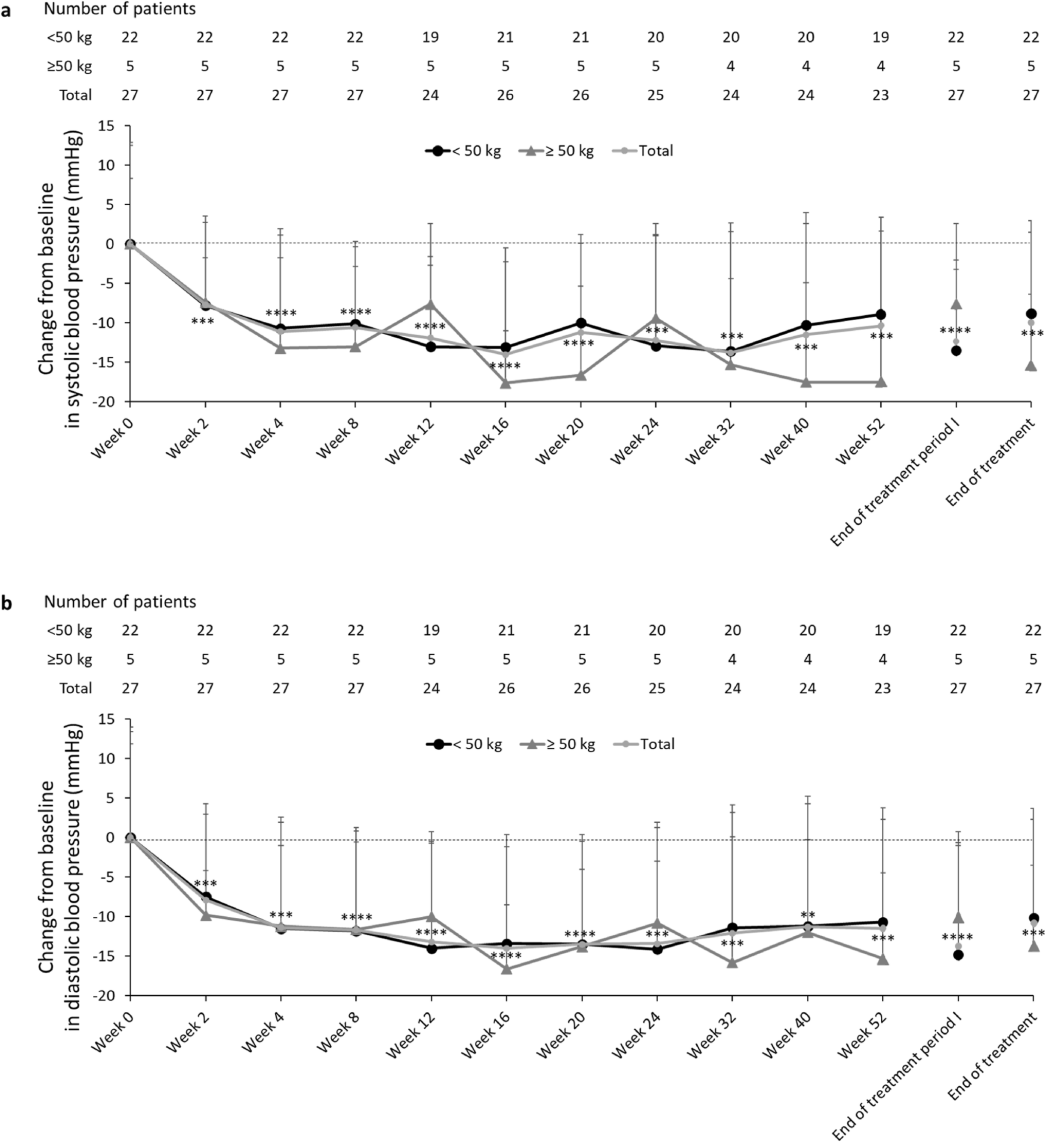
Mean changes from baseline in office through sitting SBP (**a**) and DBP (**b**) by visit for the < 50 kg group, ≥ 50 kg group and total population. At Week 0, patients weighing < 50 kg were started on an initial dose of 2.5 mg azilsartan and patients weighing ≥ 50 kg on 5 mg azilsartan. Azilsartan dose was titrated up to 5, 10 and 20 mg in patients weighing < 50 kg and to 10, 20 and 40 mg in patients weighing ≥ 50 kg. Data represent the mean and standard deviation. Nominal *p* values for the total population are shown (**p < 0.01, ***p < 0.001, ****p < 0.0001). *DBP* diastolic blood pressure, *FAS* full analysis set, *RAS* renin-angiotensin-system, *SBP* systolic blood pressure

Mean changes in SBP and DBP at each visit are plotted for each weight group and the total population (Fig. 3a and b). In the whole cohort at the end of treatment period I, the mean changes from baseline in SBP and DBP were − 12.4 and − 13.9 mmHg, respectively. By the end of azilsartan treatment, the mean changes from baseline in SBP and DBP were − 10.0 and − 10.9 mmHg, respectively. Additionally, mean changes in SBP and DBP from baseline to the end of azilsartan treatment were − 8.8 and − 10.3 mmHg in the < 50 kg group and − 15.4 and − 13.6 mmHg in the ≥ 50 kg group, respectively.

In total, 44.4% of patients achieved target BP by Week 2. At the end of treatment period I and azilsartan treatment, 63.0% (68.2% in the < 50 kg group; 40.0% in the ≥ 50 kg group) and 40.7% (36.4% in the < 50 kg group; 60.0% in the ≥ 50 kg group) of the patients achieved the target BP, respectively.

## Discussion

This study evaluated the long-term safety and efficacy of azilsartan in young patients aged 6–15 years with hypertension and revealed that azilsartan has acceptable tolerability in once-daily doses of 2.5–20 mg in patients weighing < 50 kg and 5–40 mg in those weighing ≥ 50 kg. Most patients (88.9%) experienced at least one TEAE, however, the majority (55.6%) of TEAEs were deemed to be unrelated to the study medication. TEAEs (related or unrelated to the drug) were mostly mild to moderate in severity, similar to the safety profile of azilsartan in Japanese adults [7]. One patient had three severe and serious TEAEs: kidney transplant rejection, complications of the transplanted kidney, and AKI. AKI was drug-related and also related to the patient’s underlying disease and concomitant medications according to the investigator. Laboratory tests, vital signs, physical examinations and ECG assessments showed no clinically remarkable findings except in one patient who had a slight increase in serum creatinine level. No safety concern was revealed in this study.

In a previous clinical trial in hypertensive adults [7], the mean reduction in SBP and DBP were reported as − 21.8 mmHg and − 12.4 mmHg, respectively. In the present study, 63.0% of patients met their target BP at the end of treatment period I, with a mean reduction in SBP and DBP of − 12.4 mmHg and − 13.9 mmHg, respectively. Whilst reduction in DBP in the children in our study was similar to the reduction reported in adults [7], SBP reduced to a smaller extent in the children than the adults. The reason for this observation is unknown but the greater baseline SBP in adults (160.0 mmHg) than children (125.7 mmHg) in the studies may be a contributory factor. By the end of azilsartan treatment, 40.7% of patients had achieved target BP; however, most patients who did not achieve their target BP had values close to the target values, and none had large increases in the values. In patients who did not achieve their target value, the dosage of azilsartan may not have been escalated because of the small excess over the target values. Such insufficient escalation of azilsartan’s dosage may have had a larger effect at the end of azilsartan treatment than at treatment period I. Interestingly, the reduction in BP was seen as early as Week 2, with 44.4% of patients achieving target BP at this visit. The mean changes from baseline in SBP and DBP fluctuated throughout the study, possibly because of the small sample size. Nevertheless, mean changes were consistently below 0, which suggests a persistent BP-lowering effect of azilsartan throughout the 52-week treatment period.

The main limitations of this study arise from the open-label and single-arm design of the study and the small sample size, particularly in the ≥ 50 kg group; despite enrolling patients from 34 institution sites across Japan, the target sample size was not achieved due to the low number of paediatric patients with hypertension and reluctance of the patients and their families to switch medication to participate in this study. Another limitation was that most patients had secondary hypertension and only 3 (11.1%) had essential hypertension. Furthermore, children aged < 6 years and those with poorly controlled or significant comorbidities, which impedes the extrapolation of our data to a wider population of children with hypertension.

Larger, randomised trials are needed in younger and more varied populations to reconfirm the safety and efficacy of azilsartan in children with hypertension.

## Conclusion

In conclusion, this study shows that once-daily azilsartan may be an effective antihypertensive medication with an acceptable safety profile in hypertensive children aged between 6 and 15 years. The safety and efficacy are in line with those observed in hypertensive adults at comparable weight-adjusted doses of azilsartan. Further studies with larger sample sizes would help to confirm these findings.

## Supplementary Information

Below is the link to the electronic supplementary material.

Supplementary file1 (PDF 157 KB)
