# Correction to: Safety and efficacy of azilsartan in paediatric patients with hypertension: a phase 3, single-arm, open-label, prospective study

**DOI:** 10.1007/s10157-021-02176-8

**Published:** 2022-01-13

**Authors:** Shuichi Ito, Yuya Nishiyama, Kenkichi Sugiura, Kazuaki Enya

**Affiliations:** 1 Department of Pediatrics, Yokohama City University, Yokohama, Kanagawa Japan; 2 Takeda Development Center Japan, Takeda Pharmaceutical Company Limited, Osaka, Japan

## Correction to: Clinical and Experimental Nephrology 10.1007/s10157-021-02159-9

The article “Safety and efficacy of azilsartan in paediatric patients with hypertension: a phase 3, single-arm, open-label, prospective study”, written by Shuichi Ito · Yuya Nishiyama · Kenkichi Sugiura and Kazuaki Enya was originally published Online First without Open Access. With the author(s)’ decision to opt for Open Choice the copyright of the article changed on 16th December 2021 to © The Author(s) 2021 and the article is forthwith distributed under the terms of the Creative Commons Attribution 4.0 International License (https://creativecommons.org/licenses/by/4.0/), which permits use, sharing, adaptation, distribution and reproduction in any medium or format, as long as you give appropriate credit to the original author(s) and the source, provide a link to the Creative Commons licence, and indicate if changes were made.

The original article has been corrected.

