# Supplementary material for: Safety and efficacy of azilsartan in paediatric patients with hypertension: a phase 3, single-arm, open-label, prospective study
Source: Clin Exp Nephrol. 2021 Nov 27;26(4):350–8. doi: 10.1007/s10157-021-02159-9 (PMC8930870; doi:10.1007/s10157-021-02159-9)
Supplement: Supplementary file 1 — Supplementary file1 (PDF 157 KB) [file 10157_2021_2159_MOESM1_ESM.pdf]

Supplementary Information

Online Resource TEAEs occurring in ≥5% of the total patients (SAS)

**Adverse event by SOC**	**<50 kg group (N=22)**	**≥50 kg group (N=5)**	**Total (N=27)**
Any TEAEs	19 (86.4%)	5 (100.0%)	24 (88.9%)
Gastrointestinal disorders			
Nasopharyngitis	9 (40.9%)	2 (40.0)	11 (40.7%)
Constipation	3 (13.6%)	1 (20.0)	4 (14.8%)
Influenza	3 (13.6%)	1 (20.0)	4 (14.8%)
Gastroenteritis	4 (18.2%)	0	4 (14.8%)
Epistaxis	2 (9.1%)	1 (20.0%)	3 (11.1%)
Abdominal pain	3 (13.6%)	0	3 (11.1%)
Pyrexia	3 (13.6%)	0	3 (11.1%)
Otitis media	3 (13.6%)	0	3 (11.1%)
Dizziness	3 (13.6%)	0	3 (11.1%)
Stomatitis	2 (9.1%)	0	2 (7.4%)
Sinusitis	2 (9.1%)	0	2 (7.4%)
Upper respiratory tract infection	2 (9.1%)	0	2 (7.4%)
Contusion	2 (9.1%)	0	2 (7.4%)
Ligament sprain	2 (9.1%)	0	2 (7.4%)
Serum creatinine increased	2 (9.1%)	0	2 (7.4%)
Renal impairment	1 (4.5%)	1 (20.0%)	2 (7.4%)
Asthma	2 (9.1%)	0	2 (7.4%)
Cough	2 (9.1%)	0	2 (7.4%)
Dermatitis atopic	2 (9.1%)	0	2 (7.4%)

Values are n (%).

***SAS* safety analysis set, *SOC* System Organ Class, *TEAE* treatment-emergent adverse event**
